# Case of Mollities Ossium, with Observations

**Published:** 1845-01

**Authors:** S. Solly

**Affiliations:** Assistant Surgeon to St. Thomas’s Hospital


*Case of Mollities Ossium, with Observations.* By S. Solly, Esq., F. R. S., Assistant-Surgeon to St. Thomas's Hospital.—The author of this paper relates two cases of mollities ossium in the adult which have lately come before him. The first case was that of a young woman, aged 29, in whom the disease first exhibited itself seven years previous to her death, by a fracture of the clavicle, caused merely by the exertion of lifting a stool. The disease advanced in the spinal column and skull, in which latter portion of the skeleton it was of such an active character that the membranes and substance of the brain became secondarily affected, and insanity was the consequence. When in St. Thomas's Hospital, in 1840, whither she was removed in consequence of her mental derangement, the lower extremities gave way, and depriving her of the power of walking, she was obliged to push herself about the floor upon her haunches. On the 8th April 1843, she was admitted into Hanwell, where she died in the October following. The disease had attacked almost every bone in the body.

The second case was that of a woman, aged 39, whom Mr. Solly admitted into St. Thomas's Hospital a few days before her death. In this case the progress of the disease was rather different; it commenced in the lower extremeties and attacked the rest of the skeleton subsequently. The immediate cause of death was suffocation from contracted thorax, one lung wholly impervious to the air, and the other nearly so.

Mr. Solly proposes that this disease should be distinguished from other kinds of softening of the bones, by the title of osteo-malacia *rubra et fragilis*, from the colour which the bones invariably exhibit in their interior when divided, and the fact that they almost invariably break and not bend, as in rickets. The two adjectives appear advisable, as the redness in the interior exists in the early stages of rickets, an essentially different disease, while their liability to fracture is not characteristic of this complaint alone.

From a comparison of the symptoms during life with the appearances after death, he has been led to believe that the disease is of an inflammatory character; that it commences with a morbid action of the blood-vessels, which gives rise to the severe pain in the limbs invariably attendant on it, but more especially in its commencement, and exhibits itself after death by an artificial redness of the part.

